# Electric Impedance Spectroscopy in Trees Condition Analysis: Theory and Experiment

**DOI:** 10.3390/s22218310

**Published:** 2022-10-29

**Authors:** Maxim E. Astashev, Evgeny M. Konchekov, Leonid V. Kolik, Sergey V. Gudkov

**Affiliations:** Prokhorov General Physics Institute of the Russian Academy of Sciences, 119991 Moscow, Russia

**Keywords:** electric impedance spectroscopy, plant physiology, condition monitoring, cold atmospheric plasma, plasma-treated solution, plant grafting

## Abstract

Electric impedance spectroscopy is an alternative technology to existing methods that shows promising results in the agro-food industry and plant physiology research. For example, this technology makes it possible to monitor the condition of plants, even in the early stages of development, and to control the quality of finished products. However, the use of electric impedance spectroscopy is often associated with the need to organize special laboratory conditions for measurements. Our aim is to extract information about the state of health of the internal tissues of a plant’s branches from impedance measurements. Therefore, we propose a new technique using the device and model developed by us that makes it possible to monitor the condition of tree branch tissues in situ. An apple tree was chosen as the object under study, and the dependence of the impedance of the apple tree branch on the signal frequency and branch length was analyzed. The change in the impedance of an apple tree branch during drying was also analyzed. It was shown that, when a branch dries out, the conductivity of the xylem mainly decreases. The developed technique was also applied to determine the development of the vascular system of an apple tree after grafting. It was shown that the processing of the scion and rootstock sections with the help of cold atmospheric plasma and a plasma-treated solution contributes to a better formation of graft unions.

## 1. Introduction

Electric impedance spectroscopy (EIS) is a method for analyzing the electrical properties of materials and systems by the excitation of harmonic electrical signals at different frequencies. The recorded impedance vs. frequency is then related to the physical parameters or properties of materials and systems. The cell membrane of plant cells is mainly composed of phospholipids, proteins, and sugars. The phospholipid bilayer is the main framework of the cell membrane and is an excellent electrical insulator. The cell, as a whole, can be considered as a concentric spherical capacitor [1,2], in which the solution inside and outside the membrane acts as a lining, and the lipid bilayer acts as an insulating gap. The capacitance of such a capacitor is 1 μF per 1 cm^2^ of membrane area. When plant tissues are subjected to external mechanical action, the permeability of the cell membrane, and hence the concentration of electrolytes inside and outside the cell membrane, change. This results in a change in the impedance and capacitance of the tissue.

EIS began to be used in biological systems in the 1920s [3,4]. More recent studies have proposed theories about the principles of EIS as applied to a biological system. For example, Fricke proposed an equivalent principle diagram of biological tissue [5,6,7], Schwan proposed a theory of dispersion occurring in biological tissues [8,9], and the Cole–Cole equation describing dispersions was empirically derived [10]. Some of these models use a special element called a constant-phase element (CPE) [11] to better fit EIS data to the model and to differentiate between resistive and capacitive elements in the biological tissue structure. The CPE keeps its phase constant over the entire analyzed frequency range [12], which allows some of the inhomogeneities in the electrical properties of tissues due to complex biological responses when using EIS to be explained [13,14]. Most biological tissues are composed primarily of cells and extracellular fluid. The electrical properties of tissues depend significantly on the composition and distribution of these two elements. Both intracellular and extracellular fluids contain water, electrolytes, free ions, salts, and other components; therefore, their electrical behavior is mostly resistive. However, the cell membrane surrounding the cell consists of a lipid bilayer that serves as the boundary between the intracellular and extracellular environments. Due to the presence of this double layer, the cell membrane has capacitive properties [15].

Three main areas of EIS use can be distinguished: electrical impedance tomography in medicine [16,17,18,19,20,21], assessment of product quality and safety in the food industry, and the study of plant physiology in agriculture [22,23,24,25]. When applied to plant physiology, this method is mainly useful for characterizing quantitative cellular changes. Recording both the in vivo and in vitro electrical impedance of tissues provides information on the cellular structure, fluid content, characteristics, and integrity of the intra- and extracellular parts of plant tissues.

The vascular system of plants mainly performs two functions—it delivers resources to plant organs and serves as a long-distance communication system [26,27]. Features of the vascular system of plants can also be studied using EIS. To solve these problems, the transport system can be represented as a combination of resistances and capacitors. Phloem and xylem, which are relatively conductive, are separated by a low-conductivity cambium layer. In addition, electrical impedance measurements can be carried out non-invasively on relatively young shoots, so stress factors can be detected even in the early stages of plant development. As a rule, the use of EIS is associated with the need to organize special laboratory conditions for measurements. In this paper, we propose a new method for assessing the health status of the internal tissues of a plant stem by EIS, which makes it possible to monitor the condition of trees in situ, both in the field and in greenhouses. This would allow the drying of the plant, damage to the vascular system, and the rate of healing, for example, when grafting a tree, to be determined.

## 2. Materials and Methods

### 2.1. EIS Methodology

**Figure 1:**
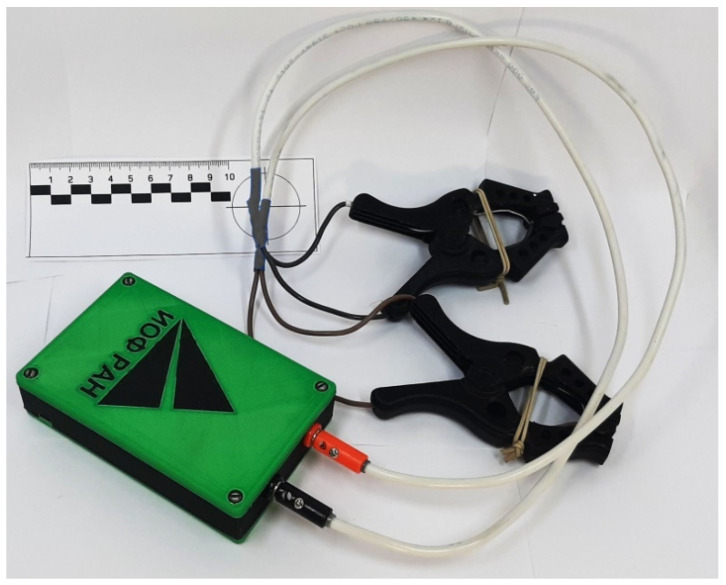
Appearance of the device for complex resistance (impedance) measurements.

**Figure 2:**
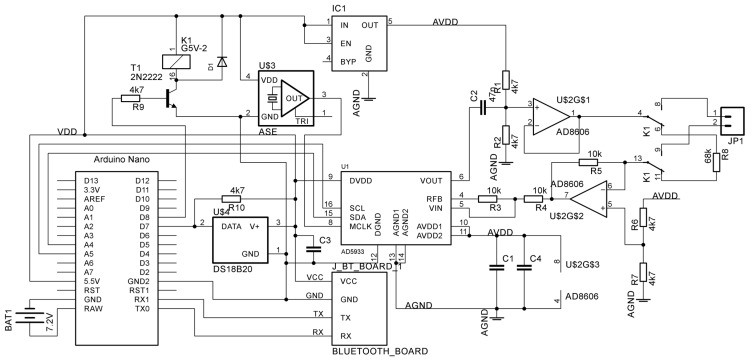
Circuit diagram of the device for complex resistance (impedance) measurements.

The complex resistance (impedance) was measured using a device (Figure 1) developed by us based on the AD5933 chip (Analog Devices) and the ATMega328 controller (Microchip) as part of the Arduino Nano V3.0 module, interfaced as standard via the I2C bus in accordance with the datasheet for the AD5933 chip (Figure 2). The interface with the computer is created by the Bluetooth module HC-05. The device is powered by two 18,650-type Li-Ion batteries connected in series. To expand the range of the measured resistances, the AD8606 operational amplifier (Analog Devices, USA) implements a voltage follower and a current-to-voltage converter in accordance with the reference design AD5933. Unlike similar devices previously described in the literature [28], the device we developed has an auto-calibration and protection function, and measurement temperature compensation function. A DS18B20 sensor (Dallas Semiconductor, Dallas, TX, USA) is used to measure temperature. The auto-calibration and protection function consists of an electromagnetic relay K1 and a 68 kOhm resistor R8. During the calibration procedure, the resistor R8 is connected to the inputs of the measurement circuit and the same frequency range scan is performed as in a routine measurement. The procedure for dividing the complex measurement results of plant samples by the complex measurement results of the resistor is implemented in the firmware of the microcontroller. We have also developed contact tongues for connecting stems with a diameter of up to 45 mm with metal contact pads composed of electrotechnical sheet nickel. The AD5933 chip outputs measurements as a pair of integers proportional to the real and imaginary components of the current through the test sample.

The measurements were carried out in order to determine the dependence of the impedance on the frequency of the voltage applied to the sample. To achieve this, a series of complex data was obtained for a number of frequencies from 2 kHz to 10 kHz with a step of 1 kHz and from 20 kHz to 100 kHz with a step of 10 kHz. Such a frequency grid made it possible, on the one hand, to make maximum use of the automation of the measurement procedure incorporated in the AD5933, and, on the other hand, saved measurement time in the frequency range from 20 to 100 kHz.

The measurement procedure consisted of a preliminary calibration of the meter with a 68 kΩ resistor, for which the input part of the AD5933 was connected to the resistor using a relay, and then the actual measurement of the test sample, in which the input part of the AD5933 was connected to the input terminals of the device using the same relay with two switching groups contacts. Accounting for calibration, the calculation of normalized data was carried out directly in ATMega328 using the formula for dividing complex numbers. The measurement result was transmitted from the controller to the computer via the RS232 protocol in the form of a table of numbers with three columns: frequency, real current component, and imaginary current component. In the field, compact portable devices were used on the Android operating system and data were transmitted via Bluetooth.

Measuring the impedance of samples of tree branches with a diameter of 6–30 mm and a length of 100–500 mm was carried out by connecting the device to the bark of the branch under study through electrolytic bridges, which consisted of rectangular pieces of hygienic cotton pads that were 10 × 30 mm in size and 2 mm thick, and wetted with 5% sodium bicarbonate solution in distilled water. The quality of the contacts was evaluated by the stability of the conductivity readings, and each sample was measured at least 5 times.

### 2.2. Grafted Tree Samples Preparation

**Figure 3:**
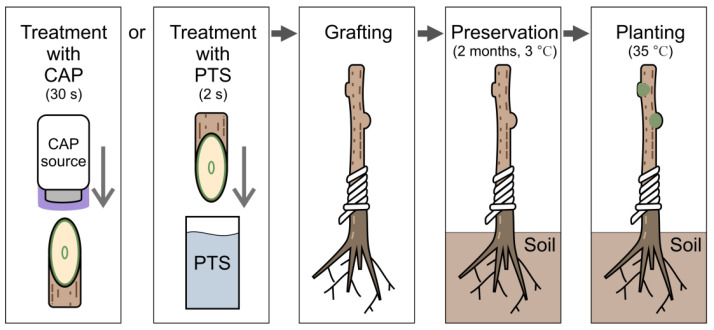
Grafted tree samples’ preparation.

We also applied the described measurement technique to determine the development of the vascular system of trees several months after grafting. Thus, it was possible to determine the quality of grafting, including in the early stages of plant development. In order to improve the quality of grafting, the cut surfaces of the scion (scion) and rootstock (rootstock) were treated with cold, atmospheric dielectric barrier discharge plasma (DBD CAP) or plasma-treated solution (PTS). After that, the rootstock–scion combinations were sent for preservation in a refrigerator. After 2 months, planting was carried out in a greenhouse, and further growth of the samples was monitored. The sample preparation protocol is shown in Figure 3.

The selection of samples for the rootstock and scion was carried out in accordance with the requirements for the quality of fruit crops GOST R 53135-2008. This study used the domestic apple tree (Malus domestica) of the melba variety. Melba is a summer apple variety first bred in Canada in 1898 from open pollinated seeds of the Mackintosh variety, and was named after the Australian singer Nellie Melba (1861–1931). The age of fruiting is 4–5 years; in dwarf rootstocks, trees begin to bear fruit in the third year after planting. It is a tree of medium height with a rounded crown, and is moderately winter-hardy. It is also early growing, with an average yield per tree of 150–200 kg.

The CAPKO mobile device developed at the GPI RAS was used as a source of CAP for the treatment of the grafting zone. The principle of its operation is described in detail in [29,30], and the method of processing the scion and rootstock samples is described in [31].

The PTS was created using a source of low-temperature plasma, which is formed by a high-frequency glow discharge in water vapor. The structure and features of this source are described earlier in the article [32]. The physico-chemical parameters of the PTS are presented in Table 1.

Based on the results obtained earlier [33,34], we chose the optimal duration of the treatment of scion and rootstock surfaces with DBD CAP using the CAPKO device, which was 30 s. The highest-quality combination of scion and rootstock during treatment with PTS was observed when the solution was diluted 5 times in distilled water.

## 3. Results

### 3.1. Impedance Measurements of Part of a Branch

EIS is an alternative to traditional methods of plant health analysis due to its high sensitivity and fast response [35]. EIS allows the properties of materials and systems to be analyzed by applying alternating electrical signals of various frequencies and measuring the corresponding electrical output signals (current or voltage). The ratio of the voltage signal to the current signal is called the impedance and depends on the frequency. The impedance can be written in rectangular coordinates (1) or in polar coordinates (2).

(1) Z=R+jX,

(2) $$Z=\left|Z\right|e^{j\varphi }\{\begin{array}{l} \left|Z\right|=\frac{\left|v\left(t\right)\right|}{\left|i\left(t\right)\right|},\\ \varphi =2\pi f\Delta t; \end{array}$$

where *R* is the active resistance and *X* is the reactance, |*Z*| is the impedance modulus, *φ* is the phase, |v(t)| is the voltage signal, and |i(t)| is the current signal. The module was calculated as the ratio of the voltage signal to the current signal, and the phase was determined by the shift between these signals.

**Figure 4:**
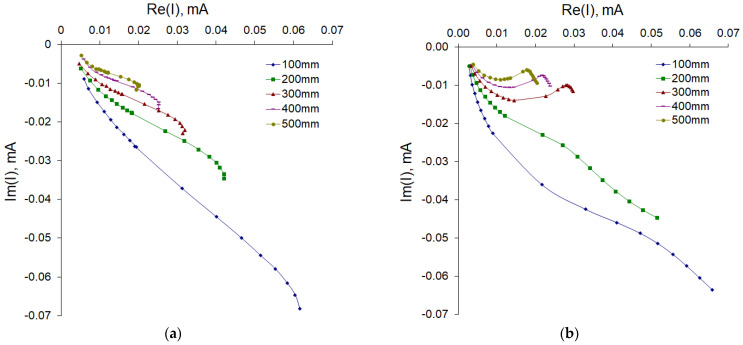
Nyquist plots for apple samples with different lengths. Experimental data (**a**) and model fitting (**b**). All samples were 23 mm in diameter. The number of samples in each experimental group is 5 and the number of measurement repetitions is 5. Data are presented as mean values.

Our method uses two electrodes to apply an alternating voltage of known amplitude to the region of interest, measure the real and imaginary components of the current in the circuit, and calculate the electrical impedance. In this bipolar system, the polarization of the electrodes can lead to a systematic error in measuring the voltage between the two electrodes. This is due to the presence of a polarization zone around the electrodes, where loads are created and where the mobility of the ions is different, as well as to partial electrolysis. As a result, spurious capacitive impedances appear at the interface between the two electrode–sample contacts, which leads to a spurious voltage drop. One way to eliminate this is to take several measurements by varying the frequency of the signal current. We obtained Nyquist plots with respect to length by measuring a branch sample with lengths of 100, 200, 300, 400, and 500 mm (Figure 4). Figure 4 shows that the graphs (with some features) corresponded to the low-frequency part of the standard plot for the high-pass RC filter. The shift of the graph along the real number axis at a frequency of 2 kHz allows the leakage resistance on the surface of the cortex to be estimated, and the main capacitive component can be estimated using the approximation of the impedance modulus, which we performed next.

**Figure 5:**
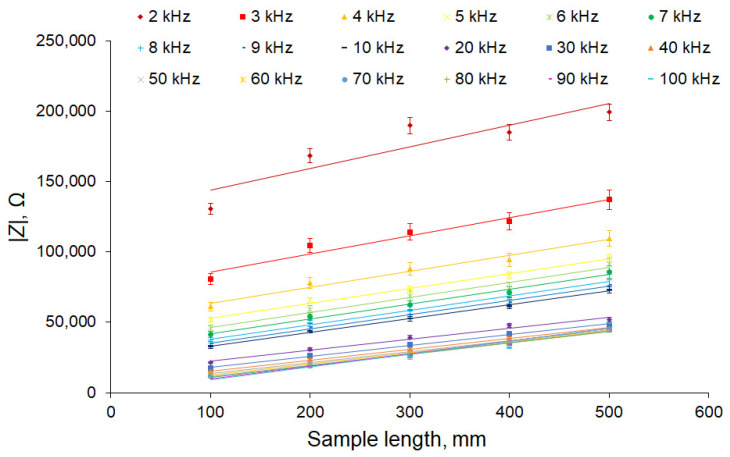
Dependence of the impedance modulus of apple samples on the sample length for different frequencies. The number of samples was 5 and the number of measurement repetitions was 5. Data are presented as mean values and standard errors.

**Figure 6:**
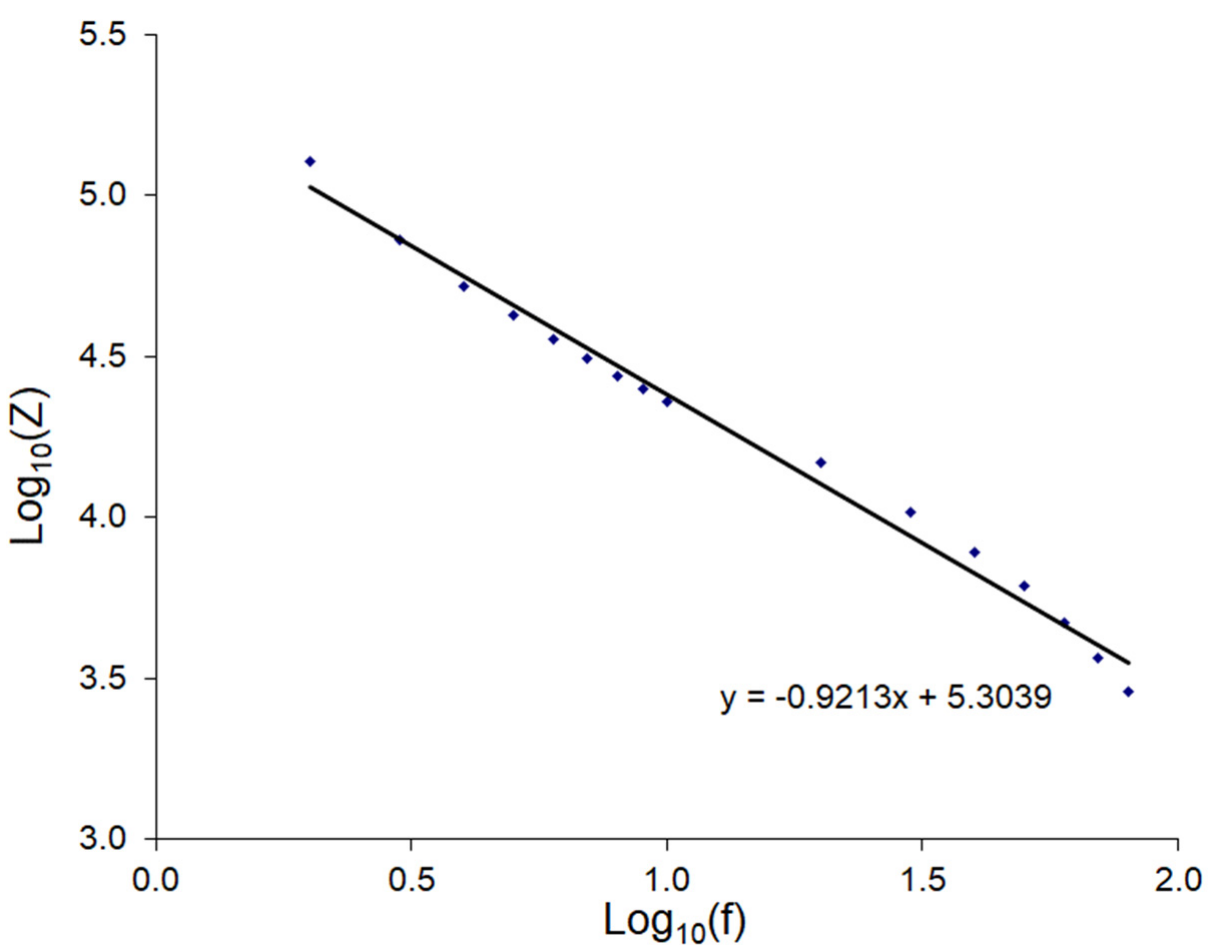
Dependence of the logarithm of the displacement of the impedance at the “zero length” of the sample on the logarithm of the frequency with linear regression.

In Figure 5, the linear dependence of the impedance modulus on the length can be seen, and the slope of the graphs, in general, was preserved for all studied frequencies; however, there was a clearly visible shift, which, for a frequency of 2 kHz, was about 120 kΩ and tended to zero with increasing frequency. If we perform a linear regression (Figure 6) for each frequency, we can obtain the value of the slope (slope) and offset at zero length (offset) (Table 2).

The regression slope of −0.9213 was very close to −1, suggesting that we were dealing with a hyperbolic dependence, meaning that the frequency dependence of impedance was mainly determined by the capacitor with a value of about 0.65 μF connected in series to the sample under study. This capacitor was formed from the contact pads of the electrodes and the insulating layer under the bark of the plant, and knowing the size of the electrode (3 cm^2^) and assuming that the dielectric constant of the wood was about 3.5 [36], and taking into account that there were two electrodes in the circuit, i.e., double layer, it was possible to estimate the thickness d of this layer by the Formula (3): *d* = 9 µm.

(3) $$C=\frac{\varepsilon_{0}\varepsilon S}{2d},$$

This capacitance can be compensated by subtracting the impedance of the form:

(4) $$Z=\frac{1}{j2\pi fC}\;,$$

**Figure 7:**
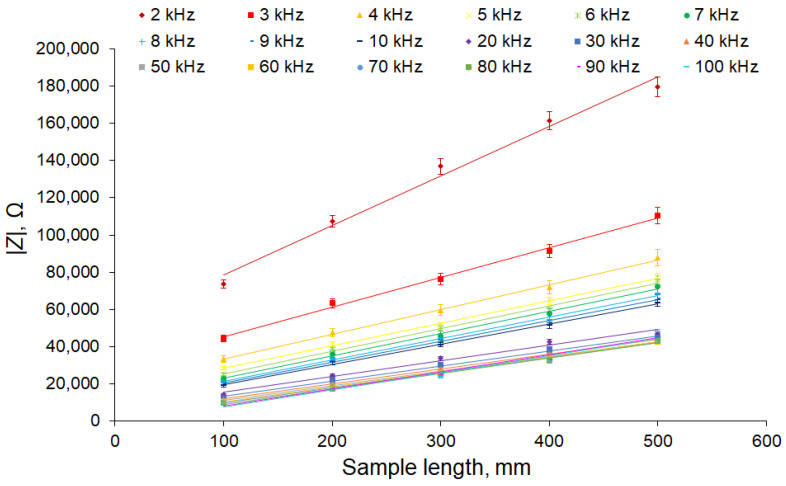
Dependence of the impedance modulus on the length of the sample for different frequencies after compensation of the capacitive transition of the electrodes. The apple tree was 23 mm in diameter. The number of samples is 5 and the number of measurement repetitions was 5. Data are presented as mean values and standard errors.

The result is shown in Figure 7. Figure 7 shows that the initial offset was almost compensated, and the graphs were in good agreement with Formula (3).

**Figure 8:**
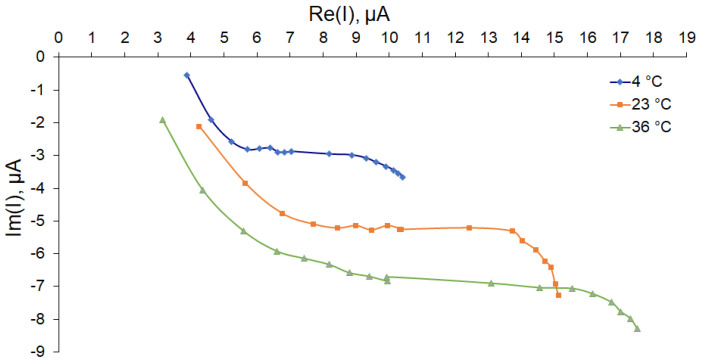
Nyquist plot for the temperature dependence of the impedance of apple nodes.

To determine the temperature dependence of the plant branch impedance, measurements at temperatures of 4, 23, and 36 degrees Celsius were taken. Thus, the outdoor conditions were simulated. The averaged data of three independent measurements of three branches 300 mm long and 12 to 16 mm in diameter are presented in Figure 8. It can be seen that the conductivity increased with increasing temperature.

### 3.2. Linear Resistance and Specific Resistance of Xylem and Phloem

**Figure 9:**
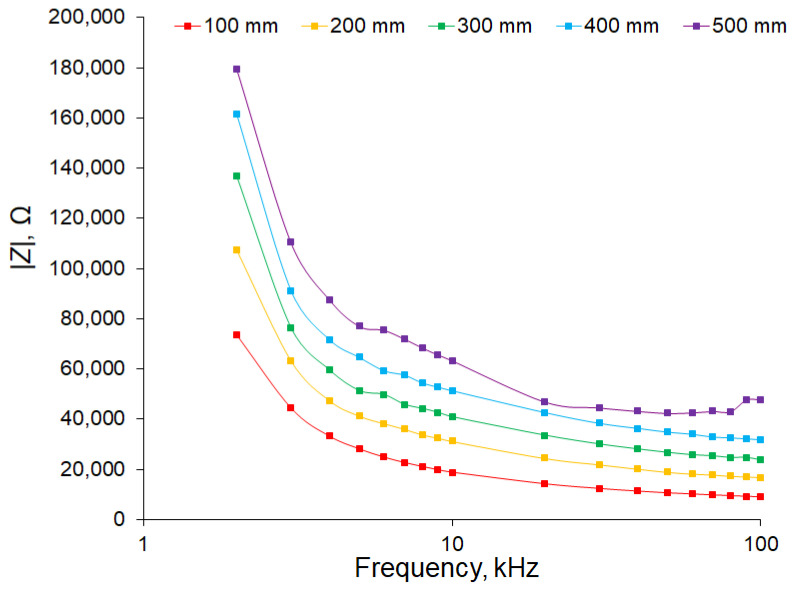
Dependence of the impedance modulus on frequency for different sample lengths after compensating for the capacitive transition of the electrodes. The apple tree was 23 mm in diameter. The number of samples in each experimental group was 5 and the number of measurement repetitions was 5. Data are presented as mean values.

To determine the conductivity of the xylem and phloem of an apple tree, we plotted the dependence of the impedance on the signal frequency, compensating for the capacitance of the contact pads of the electrodes and the insulating layer beneath the plant bark. Figure 9 shows that, at a frequency of 2 kHz, the impedance was large and further decreased asymptotically with increasing frequency. We suppose that, at low frequencies, the impedance was close to the impedance of the phloem, and as the frequency increased, the current through the insulating layer of cambium increased; at high frequencies, we could see the impedance of two parallel conductive layers: phloem and xylem.

The linear resistance at a frequency of 2 kHz was 266 Ohm/mm, and at a frequency of 100 kHz, it was about 82 Ohm/mm. Accordingly, the unit resistance of the phloem can be taken as 266 Ohm/mm, and the unit resistance of the xylem is 119 Ohm/mm.

**Figure 10:**
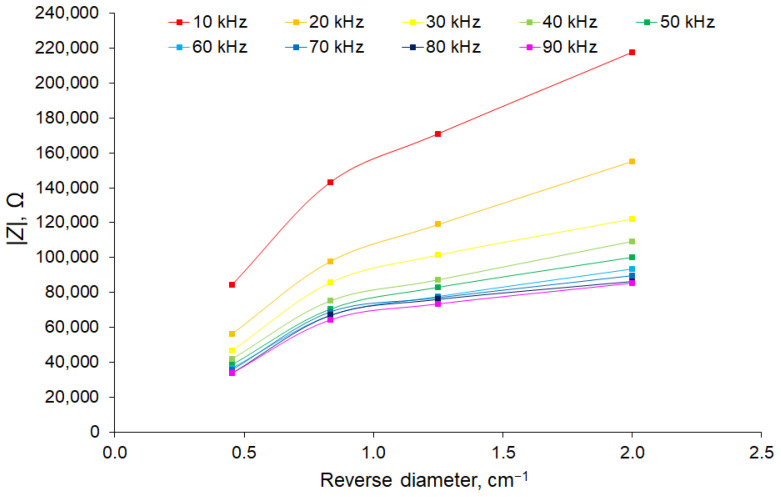
Dependence of the cambium–xylem chain impedance modulus on the reciprocal sample diameter for the high-frequency region. The number of samples in each experimental group was 5 and the number of measurement repetitions was 5. Data are presented as mean values.

To estimate the resistivity, branches of various diameters were measured. We found that the linear dependence of the impedance on the reciprocal diameter was observed only for a frequency of 3 kHz, indicating the conduction in a layer of constant thickness, the area of which increased linearly with the diameter, corresponding to the predominant conduction in the phloem. If we subtract the phloem conductivity from the total observed conductivity, we can plot the dependence of the impedance of the cambium–xylem chain on the reciprocal diameter (Figure 10).

For the cambium–xylem chain, a two-mode dependence was observed in the high-frequency region, with a break in this characteristic observed in the region of 1.2 cm^–1^. This apparently occurred as a result of the fact that small-diameter branches corresponding to the reciprocal diameter range of 1.2–2.0 cm^–1^ (0.5–0.8 cm in diameter) were non-lignified and had conductivity throughout the entire inner part of the stem up to the core. In the range of reciprocal diameters of 0.48–1.20 cm^–1^ (0.8–2.2 cm in diameter), the conductivity of the xylem was provided by a layer of constant thickness.

### 3.3. Estimating the Impedance When the Plant Dries

EIS is an alternative technology to existing methods that is already showing promising results in the agro-food industry [37,38], where it is used for a wide range of applications, such as the assessment of injury healing in fruit plants [31], grading and quality control of vegetables [39,40,41], fruit [42,43], meat [44], fish [45,46], and honey [47], as well as for pesticide detection [48] and the valorization of agro-food waste [49]. A relationship has also been established between the electrical behavior of biological tissue and changes in its properties due to freezing [50] or drying [40]. For example, it was found that, in the low-frequency region, due to the high electrical capacitance of cell membranes, electric current flowed only through the extracellular fluid, which had a relatively high resistance. However, in the high-frequency region, the impedance was greatly reduced because the current could pass through the intracellular fluid, which had a relatively low resistance. This phenomenon resulting from cellular structures in biological tissue is known as β-dispersion [51]. The impedance in the lower frequency range decreased at the early stage of potato drying [40], while, at the late stage of drying, the impedance increased markedly. This result shows a decrease in extracellular resistance and a change in the electrical capacitance of dried samples.

**Figure 11:**
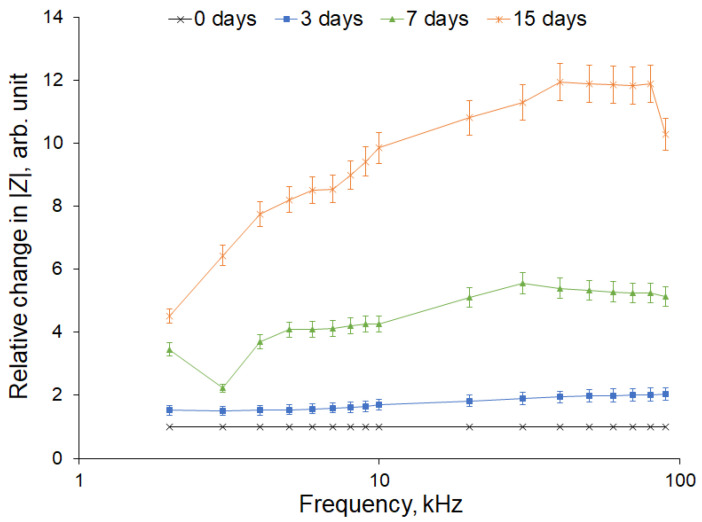
Relative change in the impedance modulus during the drying of apple tree branches for 3, 7, and 15 days. The number of samples was 10 and the number of measurement repetitions was 5. Data are presented as mean values and standard errors.

However, the change in impedance when tree branches dry out is of a different nature. We applied the developed method for monitoring the liquid content of the branches of the apple tree. The drying of branches separated from the trunk and devoid of leaves at room temperature was carried out for up to 15 days. When dried, the amount of water decreased, as evidenced by the decrease in the mass of the branch. The change in the impedance modulus depending on the frequency was non-linear: as the frequency increased, the change in the impedance also increased (Figure 11). This suggests that, when the branch dried out, the conductivity of the xylem mainly decreased.

### 3.4. Grafting Zone Impedance Estimation

**Figure 12:**
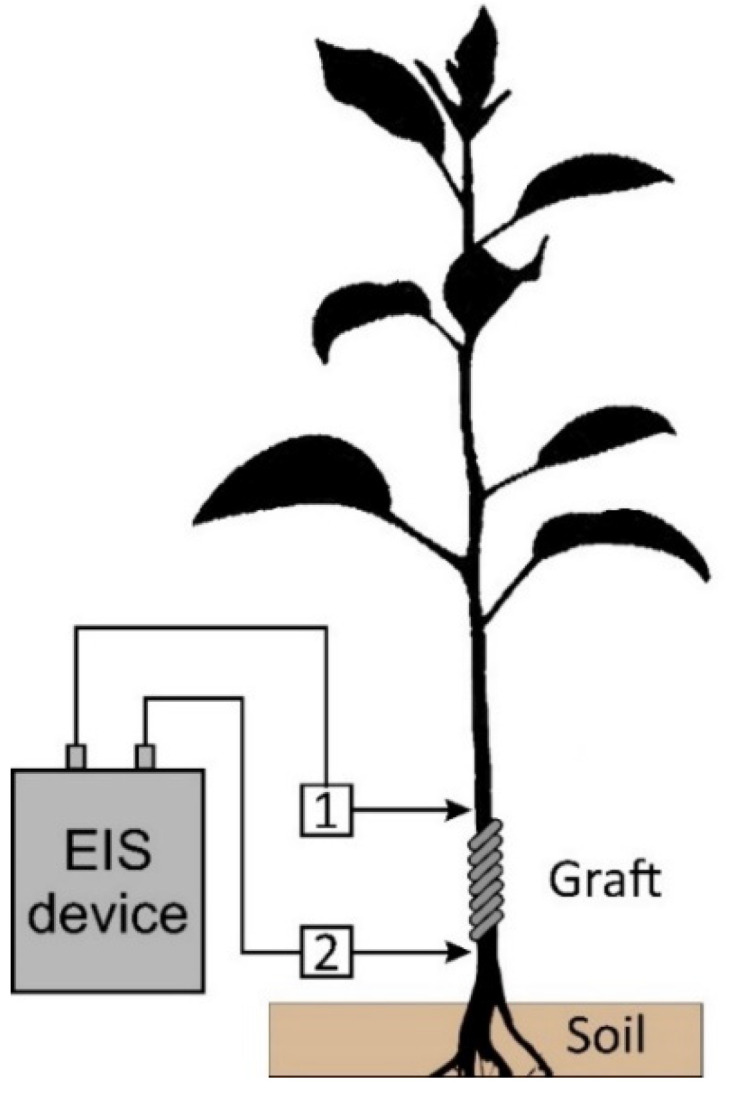
Measurement of the electrical impedance of the graft zone in an apple seedling 6 months after grafting. 1, 2—electrodes located at a distance of 80 ± 5 mm from each other.

**Figure 13:**
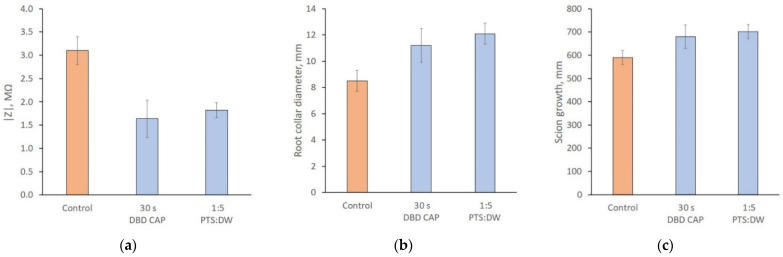
Graft zone impedance modulus after compensating for the electrode capacitive transition (**a**), the root collar diameter (**b**), and the scion growth (**c**) 6 months after grafting. Results are presented for 3 experimental groups: control, direct treatment with DBD CAP (30 s exposure duration), and indirect treatment with PTS diluted in DW with a 1:5 proportion. The number of samples in each experimental group is 30 and the number of measurement repetitions is 5. Data are presented as mean values and standard errors.

To assess the healing process of the grafting site, electrical impedance measurements were taken in the grafting zone. Measurements were taken 6 months after grafting. The electrodes were placed at a distance of 80 ± 5 mm from each other, as shown in Figure 12. The first group of samples during the grafting process was treated with CAP and the second group of samples was treated with PTS solution. The graft zone impedance modulus after compensation for the capacitive transition of the electrodes is shown in Figure 13a. As can be seen from the figure, the samples treated with CAP and PTS had a lower impedance in the scion zone, indicating a more developed vascular system. These results correlate with the difference in the biomass of the samples. Figure 13b,c shows the collar diameter and increment length at the time of the impedance measurement.

This result can be explained by a combination of effects that occur during the processing of scion and rootstock cut surfaces using CAP and PTS. The surface is activated by active forms of oxygen and nitrogen, which contribute to the lignification of the contact zone, a decrease in the surface roughness of the cuts, and an improvement in the wettability of these surfaces [52]. These effects significantly improved the adhesive properties of the sections of the grafted parts and the resistance of the grafted plant to subsequent physical stresses.

## 4. Discussion

The use of EIS in the frequency range from 100 Hz to 100 kHz makes it possible to work in the range corresponding to the end of the α-dispersion and the wide spectrum of the β-dispersion and, therefore, to effectively detect bioimpedance differences in samples. At low frequencies, the current flows through the extracellular fluid as the components of the layers of cell membranes and organelles (proteins, macromolecules, and other components) have time to polarize and thus prevent the flow of an electric current through them, acting as capacitive components. In the higher frequency range, the capacitance decreases, which reduces the electrical resistance of the tissue [20,53].

**Figure 14:**
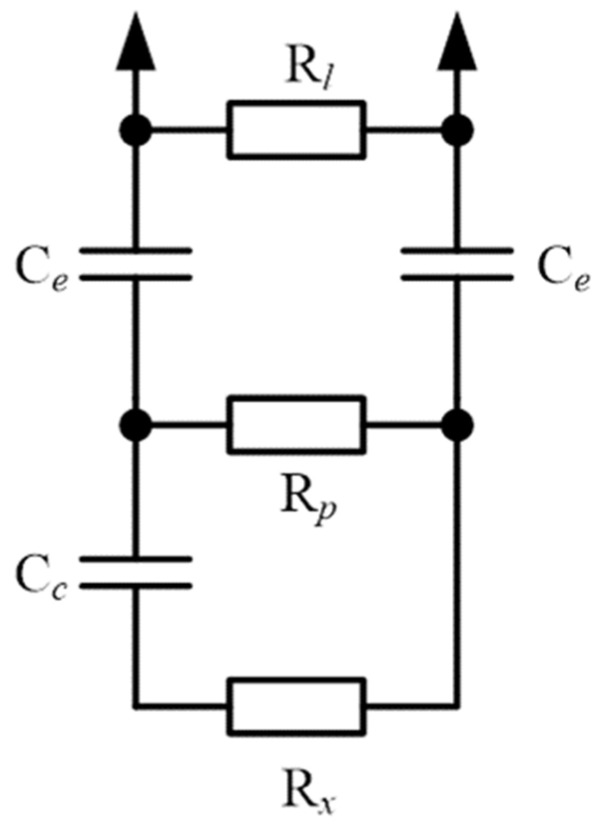
Electrical model of a section of a tree branch connected to an impedance meter. *R_l_* is the leakage resistor forming leakage current along the surface of the bark, *R_p_* is the phloem resistance, *R_x_* is the xylem resistance, *C_e_* represents the capacitors formed by electrode connection points and non-conductive layers of the sclerenchyma, periderm, and epidermis, *C_c_* is a capacitor formed by a non-conductive layer of cambium, and arrows are the points of attachment to the measuring equipment.

According to the results and the structure of plant stems, the model of a tree branch section during impedance measurement can be represented as an electrical schema (Figure 14). Here, *R_l_* is the resistance of leakage forming shift impedance along the real axis in low frequencies (2 kHz), *R_p_* is the resistance of the phloem, *R_x_* is the resistance of the xylem, *C_e_* represents the capacitors formed by the electrode connection points and non-conductive layers of the sclerenchyma, periderm, and epidermis, and *C_c_* is a capacitor formed by a non-conductive layer of cambium between the xylem and phloem.

In the simplest case, the resistance of a conductor section is determined by the formula:

(5) R=ρl/S ,

where *ρ* is the resistivity of the material, *l* is the length of the area under study, and *S* is the cross-sectional area. Thus, for phloem resistance, the formula is:

(6) $$R=\rho_{p}l/\left(2\pi Dh\right),$$

where *ρ_p_* is the resistivity of the phloem, *l* is the length of the node between the electrodes, *D* is the diameter of the node, and *h* is the thickness of the phloem. For xylem, the resistance formula is:

(7) $$R=2\rho_{x}l/\left(\pi D^{2}\right),$$

where *ρ_x_* is the resistivity of the xylem, *l* is the length of the node between the electrodes, and *D* is the diameter of the node. *R_l_*, *C_c_*, and *C_e_*, we suppose, do not depend on node geometry. The goal function for minimizing the procedure is:

(8) $$F=\underset{l,f}{\sum }\left({\left(Re\left(Z_{M}\left(l,f\right)\right)-Re\left(Z_{E}\left(l,f\right)\right)\right)}^{2}+{\left(Im\left(Z_{M}\left(l,f\right)\right)-Im\left(Z_{E}\left(l,f\right)\right)\right)}^{2}\right),$$

where *Z_M_(l,f)* is the model-estimated impedance and *Z_E_(l,f)* represents the experimental data. Goal seeking was performed with internal functions of MSExcel with the same name iteratively for all parameters (except *d*). The identified solution parameters are shown in Table 3, and the estimated impedance versus length is presented in Figure 4b. Comparing Figure 4a,b, it can be seen that the main features of the behavior of the impedance with a change in the frequency and length of the branch were reproduced. This indicates the correctness of our proposed model. In addition, the order of magnitude was the same as the capacitance of the electrodes estimated by fitting the model and by estimating the one we produced earlier by approximating the impedance modulus.

Figure 8 shows that conductivity had temperature dependence. The linear regression of temperature made it possible to estimate the rate of change in conductivity with temperature according to the formula [54]:

(9) $$\sigma \left(T\right)=\sigma_{300}\left(1+\alpha \left(T-300K\right)\right),$$

where *σ(T)* is the conductivity at temperature *T*, *σ*_300_ is the conductivity at 300 K, and *α* is the temperature dependence coefficient. When determining the slope of the linear regression in the high frequency range of 20–90 kHz (to eliminate the effect of capacitive components), *α* = 0.014–0.017 K^–1^, which coincides with the normal value for small ions (such as hydrogen and hydroxyl) [54]. Compensating for this sharp temperature dependence required the installation of a temperature probe in the device for outdoor conditions. Therefore, a DS18B20 digital sensor was installed on our device.

The dependence of the impedance modulus on frequency for different sample lengths (Figure 9) indicates that, at low frequencies, the impedance was close to the impedance of the phloem, as the frequency increased, the current through the capacitor *C_c_* increased, and at high frequencies, we could see the impedance of two parallel resistors, *R_p_* and *R_x_*.

We also carried out a study of the transport system of the apple tree on a model of a grafted plant (Figure 13). The value of the active part of the impedance characterized the development of the vascular system and the thickness of the callus tissue at the junction of the scion and rootstock. The lower the active resistance (the real part of the impedance), the better the graft union formed. As shown by previous studies on pear and cherry trees [31,55], lower values of the active part of the impedance correlated with a higher rate of plant biomass gain during the first 6 months of development. As can be seen from Figure 13, this result was also reproduced for apple samples.

The method developed by us proved to be an economical and easy-to-use alternative to traditional methods for assessing the fluid content in the trunk and branches of trees. In addition, the technique made it possible to successfully determine the degree of healing of damage in trees, the development of the vascular system, and the quality of grafting.

## 5. Conclusions

The non-invasiveness, ease of use, the possibility of field use, and the speed of obtaining information led to the active use of EIS in a wide range of human activities, in particular, in the agroindustry and in plant physiology research. In this article, we attempted to not only measure the impedance of the branches as a whole, but also to decompose the received data into components responsible for the various tissues of the branch. This was necessary to formalize the algorithm for the automatic calculation and separation of the electrical characteristics of the key components of the branch in order to determine their health status; to this end, we introduced an electrical model and fit its parameters. The developed technique was used to characterize the internal damage of trees during drying and grafting. It has been shown that, when a branch dries out, the conductivity of the xylem mainly decreased. It was also shown that the impedance of the grafting zone 6 months after grafting was significantly lower with additional processing of graft and rootstock sections using CAP and PTS. These results correlate with the biomass of the samples, indicating a more developed vascular system of the samples subjected to CAP treatment compared with the control samples.

## Figures and Tables

**Table 1:** Physicochemical properties of the PTS after treatment with high-frequency glow discharge of an aqueous solution of 0.1 M NaCl.

Exposure Time, min	Electrical Conductivity, mS/cm	[O_2_], µM	pH	Redox, mV	NO_3_−, mM	H_2_O_2_, mM
0	7.3 ± 0.5	273 ± 5	6.7 ± 0.1	303 ± 7	<0.01	<0.01
40	24.9 ± 1.2 *	261 ± 8	8.3 ± 0.2 *	598 ± 26 *	22.05 ± 0.98 *	7.12 ± 0.68 *

* Statistical differences relative to control (*p* < 0.05, Student’s *t*-test).

**Table 2:** Slope and offset of the linear function obtained by approximating the dependence of the impedance modulus on the sample length and capacitance formed by electrode connection points and non-conductive layers of the sample.

Frequency, kHz	Slope, Ω/mm	Offset, Ω	Capacitor Capacitance, F
2	153.49	128499	6.19285 × 10^−10^
3	129.49	72777	7.28962 × 10^−10^
4	113.61	52060	7.64286 × 10^−10^
5	105.13	42445	7.49935 × 10^−10^
6	106.57	35896	7.38963 × 10^−10^
7	106.01	31166	7.29526 × 10^−10^
8	102.85	27541	7.22355 × 10^−10^
9	101.32	25014	7.06959 × 10^−10^
10	98.918	22916	6.94515 × 10^−10^
20	77.757	14902	5.34005 × 10^−10^
30	76.875	10410	5.09622 × 10^−10^
40	77.063	7790.6	5.10727 × 10^−10^
50	77.07	6106.3	5.21281 × 10^−10^
60	78.695	4697.1	5.64728 × 10^−10^
70	80.11	3681.2	6.17636 × 10^−10^
80	80.873	2879.9	6.90801 × 10^−10^

**Table 3:** Model parameters.

Parameter	Value
*R_l_*, Ohm	40,000
*C_e_*, F	3.97 × 10^−10^
*ρ_p_*, Ohm·m	29.7
*Ρ_f_*, Ohm·m	19.3
*C_c_*, F	7.17 × 10^−10^
*D*, m	0.023
*d*, m	0.002

## Data Availability

Not applicable.
